# Vinegar postbiotic solutions obtained from five red fruits processed using traditional methods exhibit different biochemical properties and antimicrobial and antioxidant effects

**DOI:** 10.1002/fsn3.4459

**Published:** 2024-10-30

**Authors:** Oğuzhan Özdemir

**Affiliations:** ^1^ Department of Veterinary Science, Technical Sciences Vocational School Batman University Batman Turkey; ^2^ Central Laboratory Application and Research Center Batman University Batman Turkey

**Keywords:** antimicrobial activity, antioxidant capacity, bioactive components, postbiotics, red fruit vinegar

## Abstract

Pomegranate, hawthorn, gilaburu, blackberry, and rosehip vinegar postbiotic solutions (VPS) were produced by traditional methods. The bioactive components of VPS, antioxidant capacity, antimicrobial activities, minimum inhibition concentration (MIC), and minimum bactericidal concentration (MBC) assays were determined. While rosehip VPS has the highest amount of lactic acid, phenolic and flavonoids, gilaburu VPS has the highest butyric acid. The highest antimicrobial activities were observed for hawthorn VPS on *C. albicans* and *S. aureus*, for gilaburu VPS on *S. poona* and *S. aureus*, for blackberry VPS on *C. albicans* mold and *S. agalactiae*, for pomegranate VPS on *E. coli* and *S. agalactiae* and for rosehip VPS on *C. albicans* and *S. agalactiae*. Moreover, the mortality values were reported as MBCs: hawthorn for *S. aureus* (94.6% at 0.03 mg mL^−1^) and *S. paratyphii* A (94.1% at 0.03 mg mL^−1^), gilaburu for *S. aureus* (93.4% at 0.06 mg mL^−1^) and *P. aeruginosa* (93.2% at 0.13 mg mL^−1^), rosehip for *S. agalactiace* and *E. coli* (93.7–91.7% at 0.06 mg mL^−1^), pomegranate for methicillin‐resistant *S. aureus* (96.0% at 0.5 mg mL^−1^), and blackberry for *S. aureus* (91.3% at 0.25 mg mL^−1^) and *P. aeruginosa* (92.1% at 0.13 mg mL^−1^), in addition to an equal mixture of the five VPSs for *S. aureus* and *P. aeruginosa* (85.6% at 0.06 mg mL^−1^). The MICs for VPS were generally found in a 0.5 mg mL^−1^dilution of each vinegar. Remarkably, common and local fruits can be rich sources of bioactive components without the need for imported products or expensive processing methods or equipment. This study demonstrated that rosehip VPS has the greatest potential as both a nutrient and a natural disinfectant.

## Graphical abstract

The study finds that rosehip vinegar postbiotic solutions (VPS) has the highest amounts of lactic acid, phenolics, and flavonoids, making it a strong nutrient and disinfectant. Different VPS types show varying antimicrobial activities: hawthorn and rosehip are effective against *Candida albicans*, while gilaburu excels against S. aureus. Mortality values (MBCs) for different VPSs vary, with notable efficacy at specific concentrations. The research highlights the potential of common fruits as rich, cost‐effective sources of bioactive compounds.

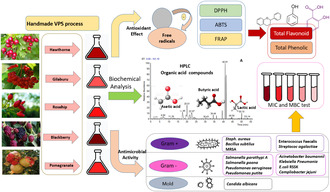

## 1 INTRODUCTION

For centuries, vinegar has been used in numerous applications such as traditional foods, medicines, and disinfectants for various ailments because of its postbiotic properties and delicious taste. The earliest known report of vinegar dates back over 2000 years, according to which Hippocrates (approximately 420 BC) used vinegar for treating wounds. Currently, vinegar is considered a “superfood” that can lower blood pressure in humans, provide antioxidant defense, slimming, aid digestion, and improve skin quality (Launholt et al., 2020; Yagnik et al., 2018).

Produced by food‐grade microorganisms, postbiotics are used in industry for biopreservation, food packaging purposes, prevention and control of foodborne biofilms, and to provide various benefits to the consumer (Moradi et al., 2020). According to The International Scientific Association of Probiotics and Prebiotics, postbiotics are defined as “inanimate microorganisms and/or their components that confer a health benefit on the host” (Salminen et al., 2021). During the growth of probiotics, soluble factors (biomolecules or metabolic by‐products) are secreted by bacteria (probiotic or non‐probiotic) or released after bacterial degradation (Homayouni Rad et al., 2021). When vinegar fermentation is completed, the supernatant is obtained by filtration, which is called the cell‐free supernatant (CFS) or vinegar postbiotic solution (VPS). The CFS of vinegar produced from fruits or vegetables contains multiple bioactive compounds, each of which is a postbiotic component (Aguilar‐Toalá et al., 2018).

Fruit fermentation produces a bioliquid containing various functional molecules such as organic acids, polyphenols, melanoidins, and tetramethylpyrazine (Ousaaid et al., 2021). Although vinegar has various components, the main ingredient is acetic acid (4%–8%; Shishehbor et al., 2017). Moreover, vinegar can be used as an antimicrobial resource (Park et al., 2016). The main reason for this effect may be that vinegar affects cell membrane function, destroying transmembrane proton motility, as well as other factors such as inhibition of enzyme activity, energy competition, and inhibition of bacterial protein expression (Liu & Hannig, 2020).

The five fruits used in this research were chosen because of their postbiotic components and intense phenolic and flavonoid content, supplied fresh from five different provinces in Turkey (Belabdelli et al., 2022; Grochowski et al., 2019; Igual et al., 2022; Kajszczak et al., 2020; Laurindo et al., 2022). The fruits used in the study were chosen because of their abundance, low cost or easy production, low water requirements, market popularity, and/or five provinces representing different regional climates or environments.

Hawthorn, *Crataegus monogyna*, is widely used in traditional medicine as a herb that constitutes a valuable source of bioactive phytochemicals and bio‐nutrients. Five flavonoids are measured in hawthorn, namely vitexin2‐O‐rhamnoside, vitexin, isovitexin, rutin, and hyperoside. The major compound in the ethanol extract of hawthorn flowers in Turkey is rutin, which provides significant antioxidant activity (Belabdelli et al., 2022). Gilaburu, *Viburnum opulus* has traditionally been used to prevent ailments such as cough, cold, tuberculosis, rheumatic pains, ulcers, liver disease, diabetes, hypertension, and some stomach and kidney problems. Gilaburu is also used for the treatment of stomach or uterine bleeding and hemorrhoids. The results of published in vitro studies indicate antimicrobial, antidiabetic, antiobesity, and anti‐inflammatory effects (Kajszczak et al., 2020). Rosehip, *Rosa canina*, is a nutrient for centuries because it contains bioactive compounds, valuable pseudo‐fruits, and pharmaceutical components. Its beneficial health effects are related to its rich content of flavonoids, carotenoids, fatty acids, and vitamins (Igual et al., 2022). Pomegranate, *Punica granatum*, can be considered a multipurpose medicinal and dietary plant. The anti‐inflammatory and antibacterial properties of pomegranate are well known, and these effects play a role in the prevention and treatment of various human diseases. For example, it has been used for its neuroprotective and cardiovascular protective properties as well as its anti‐inflammatory, antioxidant, and antitumor effects. Pomegranate consumption is also associated with metabolic and antidiabetic effects (Laurindo et al., 2022). Finally, blackberry, *Rubus caesius*, is a perennial shrub known for its resistance to cold climates. Blackberries have been used as hypoglycemic, anti‐diarrheal, and anti‐inflammatory agents. Furthermore, blackberry has moderate antiproliferative potential against two colorectal carcinoma cell lines, antidiabetic activity in alloxan‐treated mice, and anti‐aggregative effect on isolated platelets (Grochowski et al., 2019).

Owing to the antioxidant potential of anthocyanins and large amounts of phenolic compounds, red fruits have beneficial effects on the physical and mental health of humans (da Cunha et al., 2016). However, in general, there are few reports comparing the antimicrobial effects and antioxidant compounds of VPSs obtained from different fruits (Antolak et al., 2021). In this study, the total phenolic, flavonoid content, and antioxidant capacities (1,1‐diphenyl‐2‐picrylhydrazine [DPPH], ferric reducing antioxidant power [FRAP], 2,2′‐azino‐bis(3‐ethylbenzothiazoline‐6‐sulfonic acid [ABTS])) were determined. Hawthorn, gilaburu, rosehip, pomegranate, and blackberry VPSs obtained from red fruits, collected from different regions of Turkey and processed using traditional methods. In particular, it was aimed to investigate the low cost and potential health benefits of VPS production against classical drugs.

## 2 MATERIALS AND METHODS

### 2.1 Chemicals and reagents

Folin–Ciocalteu reagent, sodium carbonate (Na_2_CO_3_), gallic acid, rutin, BHA, ascorbic acid, sodium nitrite (NaNO_2_), aluminum chloride (AlCl3), methanol, sodium hydroxide (NaOH), potassium persulfate (K_2_S_2_O_8_), potassium phosphate (KH_2_PO_4_), sodium acetate, hydrochloric acid (HCl), 2,3,5‐Triphenyltetrazolium chloride (TPTZ), iron chloride (FeCl_3_), FRAP, ABTS, and DPPH were obtained from Sigma‐Aldrich GmbH (Sternheim, Germany).

### 2.2 Vinegar materials

The fruits used in this study were obtained from different regions in Turkey during their harvest periods. The origins of the fruits are given in Supplementary Material 1. Moreover, vinegar mother for acid fermentation and *Saccharomyces cerevisiae* (*S. cerevisiae*) for alcohol fermentation were purchased from local markets and used for VPS.

### 2.3 Vinegar postbiotic solution production

Vinegars were produced according to the Kirci (2017) method (Kirci, 2017). Alcoholic fermentation was carried out by adding 0.25 g L^−1^ *S. cerevisiae* to the juice collected from the fruits by pressing. Alcohol fermentation was terminated when the alcohol content reached 7%. Then, 5% mother of vinegar was added to the fruit solutions for acid fermentation. The pomegranate solution was brought into contact with air to reduce the alcohol content and speed up fermentation. Fermentation was terminated when the alcohol content of the solutions fell below 0.5% from the formation of vinegar. Fermented products were converted into vinegar using these processes. We then modified the method used by George‐Okafor et al. (2020) Vinegars were centrifuged at 5000 rpm (Thermo Fisher Scientific, USA) for 30 min and filtered with a sterile 0.22 μm pore size (Millipore, Germany), yielding crude CFS (George‐Okafor et al., 2020).

### 2.4 Biochemical analysis methods

Ascorbic acid was used as a standard in the antioxidant analysis, which was performed using a Multiskan™ FC Microplate Photometer (Thermo Fisher, USA). Before starting the analysis with vinegar samples, pomegranate, hawthorn, gilaburu, and blackberry vinegars were mixed with distilled water 10 times, and rosehip vinegar was diluted 40 times. No dilution was performed for any sample in the DPPH analysis alone.

#### 2.4.1 1,1‐Diphenyl‐2‐picrylhydrazine free radical scavenging activity

The method suggested by Benvenuti et al. was used for the determination of antioxidant activity based on DPPH (Benvenuti et al., 2004). This method involves measuring the reduction in color resulting from the inhibition of the DPPH radical at a wavelength of 517 nm. Samples were taken in different volumes (0.5–100 μL), 300 μL of 1 mM DPPH radical was added, and the final volume was made up to 3 mL with methanol. The absorbance values of the samples incubated for 15 min at room temperature in the dark, were recorded using a UV–Vis spectrophotometer at a wavelength of 517 nm (Kaya et al., 2023). The results of ascorbic acid (at 0.2 μg μL^−1^ concentration) were calculated as ascorbic acid equivalents using the standard graph equation.

#### 2.4.2 The ABTS method for determining antioxidant activity

ABTS radical scavenging activity was determined with minor modifications according to the method reported by Re et al. (1999). First, 4.0827 g of KH_2_PO_4_ (potassium phosphate) was weighed and dissolved in distilled water. The pH was then adjusted to 7.4 with NaOH, and the final volume was made up to 200 mL with distilled water. Next, 100 mL of the prepared buffer was reserved for ABTS and dissolved by adding 0.1097 g ABTS. Finally, 0.066 g of K_2_S_2_O_8_ (potassium persulfate) was added to the solution and dissolved. The prepared ABTS solution was covered with aluminum foil and incubated for 12 h while stirring with a magnetic stirrer. Pipetting was performed in 3 repetitions using a 96‐well plate. A sample‐free mixture was used as the control. After 15 min of incubation, absorbance values were read at 734 nm wavelength using the Multiskan device with a 96‐well microplate.

#### 2.4.3 Ferric reducing antioxidant power

The reducing force of ferric ions (Fe^+3^) to ferrous ions (Fe^+2^) was determined according to a previously reported method (Benzie & Szeto, 1999). First, 0.3 M Na‐acetate buffer (pH: 3.6) was prepared. Then, 40 mM HCl was prepared, and 100 mL was used for TPTZ preparation. Finally, 10 mM TPTZ was dissolved in 100 mL of 40 mM HCl, and 20 mM FeCl_3_ was prepared.

##### Preparation of the FRAP reagent

The reagent was formed from 10 volumes of 0.3 M Na‐acetate buffer, 1 volume of 10 mM TPTZ, and 1 volume of 20 mM FeCl_3_.6H_2_O solution. Butylated hydroxyanisole (BHA) was prepared at a concentration of 1 μg μL^−1^ as a standard. Samples were loaded into a 96‐well microplate in 3 replications. Then, 225 μL FeCl_3_, 225 μL FRAP reagent, 25 μL sample, and standard solution in different volumes were taken, and the final volume was completed with 500 μL buffer solution and loaded into eppendorf tubes. After mixing with a vortexer and incubating for 10 min, 300 μL was transferred to the wells, and absorbances were measured at 593 nm.

#### 2.4.4 Total phenolic content

The determination of the total amount of phenolic substance is generally performed by measuring the absorbance of the blue color formed by the reduction of the Folin–Ciocalteu reagent (Slinkard & Singleton, 1977). The color intensity formed is directly proportional to the phenolic substance concentration, and the total amount of phenolic substance can be calculated. Using this method, 0.5 N Folin–Ciocalteu reagent and 10% concentration Na_2_CO_3_ were prepared. Then, 30 min after pipetting, the absorbance was read at 760 nm. Gallic acid, a phenolic compound, was used to prepare the standard graph. Different concentrations of gallic acid, 1–0.031 mg mL^−1^ (50% percent to each dilution), were prepared with methanol, and their absorbances were read. A graph of absorbance versus concentration was constructed, and the total phenolic contents of the samples were determined as gallic acid equivalents.

#### 2.4.5 Total flavonoid

The determination of total flavonoids was achieved by observing pink color formation, which is directly proportional to flavonoid concentration. In this method, 10% AlCl_3_ was prepared in a fume hood. NaNO_2_ and 1 N NaOH were prepared at 5% concentrations. Absorbance was read at 510 nm 15 min after pipetting.

Rutin was used in the preparation of the standard chart. Different concentrations of the rutin standard were prepared with methanol, and their absorbances were determined. A graph of absorbance versus concentration was constructed, and the total flavonoid amounts of the samples were determined as rutin equivalents (Park et al., 2008).

### 2.5 Organic acid analyses

This analysis was conducted at the Siirt University Science and Technology Application and Research Center. Organic acid analysis conducted by high‐performance liquid chromatography (HPLC) was used in accordance with our laboratory conditions by modifying the method reported by Shirai. First, 0.5 mL of VPS was diluted 1 to 1 with 10 mM K‐phosphate buffer (pH 2.4), passed through a microporous filter (0.22 μm), and transferred to vials (Shirai, 2022). An HPLC‐DAD (Thermo/DIONEX Ultimate 3000 series, Thermo Fisher Scientific, USA) apparatus was used for the quantitative analysis of organic acids, with a reverse phase column (C‐18 Inertsil ODS‐3 column, 5 μm particle, 4.6 × 250 mm ID). Mobile phase A (10 mM K‐Phosphate pH 2.4) and mobile phase B (acetonitrile) were used for chromatographic separation. The column flow rate was set to 1 mL min^−1^ and the column temperature was set to 28°C throughout the analysis. The sample injection and standard injection volumes were set to 50 and 20 μL, respectively. During elution, changes were made to obtain gradients of 90% A and 10% B (1 min, 1 mL min^−1^), 90% A and 10% B (12 min, 0.75 mL min^−1^), and 25% A and 75% B (0.75 min, 35 mL min^−1^). Chromatograms were analyzed at a wavelength of 210 nm. The experiments were repeated three times.

### 2.6 Antimicrobial activity

The antimicrobial effects of VPSs on indicator microorganisms were investigated using well diffusion agar and MIC assays performed according to a previously reported method (Ozdemir et al., 2022, 2023). The effect of the vinegar samples was evaluated for gr‐positive bacteria (*S. aureus* ATCC29213, *B. subtilus* B354, and methicillin‐resistant *S. aureus* (MRSA), and *E. faecalis*) and gr‐negative bacteria (*E. coli* RSSK09036, *P. aeruginosa* ATCC27853, *S. parathypi* A NCTC13, *S. poona* RM2350, and *C. jejuni* ATCC33560). Mueller‐Hinton Agar Merck 1.05437 was used for agar tests, and Mueller‐Hinton Broth (Merck 1.10293) was used for the MIC assay.

#### 2.6.1 Agar well diffusion test

Indicator microorganisms were stored at temperatures below 5°C. They were removed and reactivated in tryptic soy broth at 37°C for 18 h. Then, 12 mL of Mueller‐Hinton Agar (cooled to 50°C after autoclaving) was poured into 90 mm Petri dishes, and 1 mL of fresh culture indicator bacteria was added with a density of 0.5 McFarland and left to dry at room for 30 min. Next, 6 mm diameter wells were drilled in frozen agars, and 100 μL of each sample VPS was added to the wells and incubated overnight at 4°C. The Petri dishes were then incubated under optimal conditions. The diameters of the inhibitory zones were measured in millimeters. The Mueller‐Hinton Agar standard procedure developed by Stella and Marin was used to eliminate or reduce variability in this test method (Stella & Marín, 2009). The procedure was adopted by the Clinical and Laboratory Standards Institute (CLSI, formerly NCCLS) as a consensus standard.

#### 2.6.2 Minimum inhibition concentration test

MICs were measured using the spectrophotometric microdilution method for red VPS against indicator microorganisms at 600 nm in a 96‐well plate reader (Multiskan™ Go Microplate Reader, Thermo Scientific, USA), where 150 μL of dual‐strength Mueller‐Hinton Broth and dual‐strength vinegars were added to the first wells, and then dilutions (1:1 v/v) were added to the other wells. Then, 30 μL of bacterial suspension of fresh culture indicator bacteria with a density of 0.5 McFarland was mixed into the prepared plates and incubated at optimal temperatures for 18–24 h. Next, sample density was detected using the plate reader, and MIC was expressed as the highest dilution that inhibited growth (turbidity max in the tube is low). Among these MIC dilutions, those with positive detection of nonviable cells >99% in the medium were considered minimum bactericidal concentrations (MBC; Brauner et al., 2016).

### 2.7 Statistical analysis

For statistical analysis, GraphPad (Software Inc., San Diego, California, USA) was utilized. An analysis of variance (ANOVA) was used to assess group differences. This was followed by additional ANOVA measurements and either the Tukey's HSD test. Throughout the entire analysis, a p‐value of less than 0.05 was deemed significant.

## 3 RESULTS AND DISCUSSION

### 3.1 Biochemical analysis

#### 3.1.1 Phenolic and flavonoid amounts

The phenolic compositions and antioxidant activities of VPSs have been determined in various studies. It has been reported that the effects of VPSs on the human body are related to the phytochemical compositions and concentration, which provide beneficial biological activities (Kadiroglu, 2018). In the current study, rosehip VPS was found to have the highest lactic acid content, and the highest amount of acetic acid and butyric acid was found in gilaburu VPS.

**FIGURE 1:**
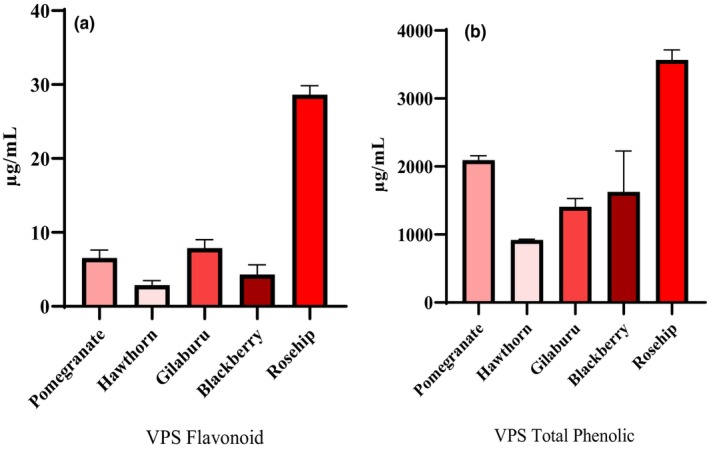
Total flavonoid (panel a) and phenolic (panel b) amounts of the VPSs obtained from five red fruits. Rutin and gallic acid were used as standards for flavonoids and phenols, respectively.

The flavonoid amounts obtained from the five VPSs are shown in Figure 1a. The highest amount of flavonoid, specifically rutin, was found in rosehip VPS, with 28.6 μg mL^−1^ (all groups, *p* < .0001). The other flavonoid contents were 7.9, 6.5, 4.3, and 2.9 μg mL^−1^ in gilaburu (hawthorn *p* < .01; blackberry *p* < .05; pomegranate *p* > .05), pomegranate (hawthorn *p* < .05; blackberry *p* > .05), blackberry, and hawthorn (blackberry *p* > .05) VPSs. The phenolic contents of the VPSs are shown in Figure 1b. The highest value, obtained as gallic acid, was detected in rosehip VPS, at 3567.7 μg mL^−1^ (pomegranate *p* < .001, other groups, *p* < .0001), and the other values were between 2094.0–923.2 μg mL^−1^.

#### 3.1.2 Antioxidant capacity

**FIGURE 2:**
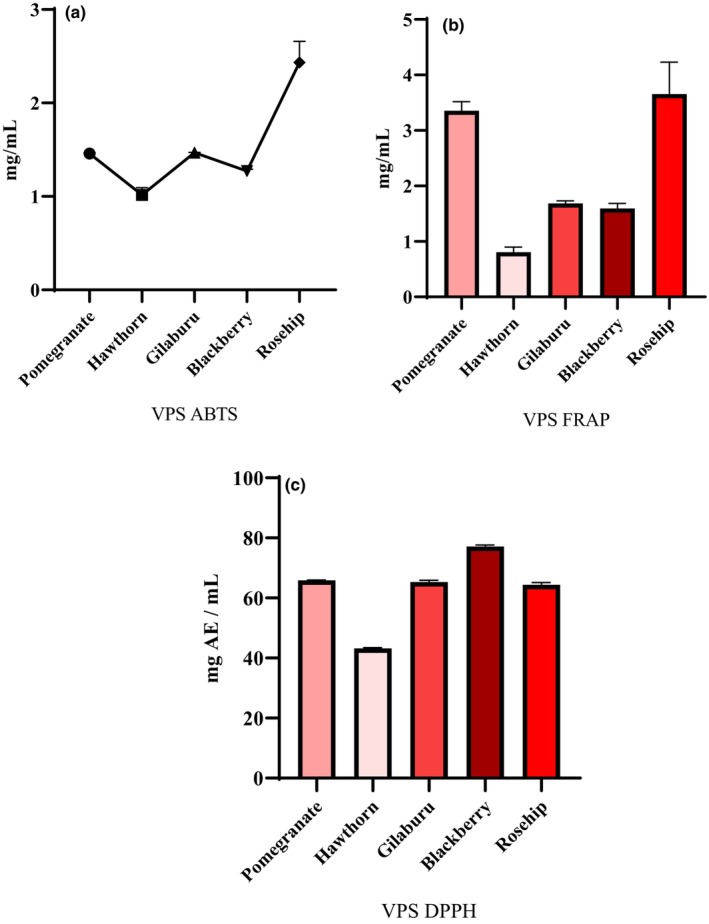
Antioxidant capacity (mg mL^−1^) of VPS for ABTS (panel a), FRAP (panel b), and DPPH (panel c) determinations (Absorbance). BHA was used as the standard for ABTS and FRAP, and ascorbic acid was used as the standard for DPPH.

The polyphenols and organic acids in fruit vinegars may contribute to their antioxidant activities, flavors, and health effects. The strong antioxidant effect of vinegar is attributed to its bioactive compounds, including carotenoids and phytosterols, as well as phenolic compounds represented by flavonoids, tannins, anthocyanins, and phenolic acids (Liu et al., 2019). Antioxidant capacities are generally associated with the amounts of phenolic and flavonoid substances in the fruit (Kawa‐Rygielska et al., 2018). In the present study, the total flavonoid and phenolic contents of rosehip vinegar were found to be higher than those of other vinegars. However, rosehip vinegar had the highest ABTS and FRAP values compared with other vinegars, whereas the highest antioxidant capacity was found in pomegranate vinegar in the DPPH analysis. ABTS antioxidant activities are shown in Figure 2a. From the analyses, the antioxidative properties in all VPSs were determined. The highest ABTS, obtained as butyl hydroxy anisole (BHA), was detected in rosehip VPS, with 2.43 mg mL^−1^, and the lowest value of 1.01 mg mL^−1^ was detected in hawthorn VPS.

The FRAP antioxidant activity is shown in Figure 2b. The highest FRAP activity, given in BHA, was detected in rosehip VPS, with 3.65 mg mL^−1^. The other activities were 3.35, 1.68, 1.59, and 0.80 mg mL^−1^ in pomegranate, gilaburu, blackberry, and hawthorn VPSs.

DPPH antioxidant activity is shown in Figure 2c. The highest DPPH obtained in terms of ascorbic acid was detected in pomegranate VPS, with 13.17 mg mL^−1^. The other VPSs exhibited values ranging from 3.260.78 mg mL^−1^.

#### 3.1.3 Organic acids

Organic acids in the vinegar structure passively diffuse through the bacterial cell wall and neutralize pH by dissociating into anions and protons. The release of protons causes the internal pH to drop, which exerts inhibitory effects on bacteria (Lingham et al., 2013). The organic acid contents of the Table VPS samples are listed in Table 1. The highest amount of lactic acid was found in rosehip VPS, at 1,819,172 ppm, followed by gilaburu, pomegranate, hawthorn, and blackberry VPSs, which varied between 1688.35 and 101.63 ppm. The highest amount of acetic acid was found in gilaburu VPS, at 126,583.98 ppm, and the acetic acid in the other VPSs varied between 30.000 and 34,885 ppm. The highest amount of butyric acid was 2700.75 ppm in gilaburu VPS, followed by hawthorn, blackberry, rosehip, and pomegranate (1198.46–216 ppm). The antimicrobial potential of the postbiotic has mainly been associated with organic acids such as lactic and acetic acids (Toushik et al., 2023).

**TABLE 1:** Analysis of organic acids by HPLC in VPS samples.

Red fruit VPS										
Organic acid	Pomegranate		Hawthorn		Gilaburu		Blackberry		Rosehip	
	RT	Amount	RT	Amount	RT	Amount	RT	Amount	RT	Amount
Lactic acid	3.46	1037.19	3.27	967.69	3.21	1688.35	3.17	101.63	3.37	1819.17
Acetic acid	3.66	3229.33	3.61	30329.50	3.61	126583.98	3.62	34855.00	3.62	32176.11
Butyric acid	9.03	216.57	9.18	1198.46	9.06	2700.75	9.05	813.40	9.05	745.117

mAU	Height	Area	Height	Area	Height	Area	Height	Area	Height	Area
Lactic acid	268.216	13.389	139.975	12.492	89.654	5.033	32.321	1.312	441.198	23.483
Acetic acid	780.994	34.300	2264.525	322.143	2379.906	310.509	2532.785	370.210	2115.365	341.756
Butyric acid	15.566	3.536	78.448	19.569	35.908	10.184	78.985	13.281	51.631	12.167

*Note*: The original peak images are given in Supplementary Material 2.

Abbreviation: Amount, the samples were measured in ppm; mAU, milli absorbance unit; RT, retention time.

#### 3.1.4 pH levels

The pH levels of the VPS samples are listed in Supplementary Material 2. The pH levels of the VPS samples were determined as pomegranate, hawthorn, gilaburu, blackberry, rosehip, and VPS mix values, 3.39, 3.25, 3.35, 3.11, 3.22, and 3.26.

### 3.2 Antimicrobial activity

The antimicrobial potential of the postbiotic has mainly been associated with organic acids such as lactic and acetic acids (Toushik et al., 2023). Ozturk et al. observed varying levels of antibacterial activity in their analyses of traditional and industrial vinegar samples (Ozturk et al., 2015). VPSs are effective antimicrobial agents against various foodborne substances and pathogenic strains, such as *K. pneumoniae*, which causes community‐acquired infections, and *E. coli*, *B. cereus*, *S. typhi*, *P. aeruginosa*, and *S. aureus*, which are responsible for diarrhea. The antifungal activity of vinegar has been tested against various fungal strains of *Aspergillus*, *Fusarium*, and *Candida*. As a result, the acetic acid content in vinegar makes it effective for treating various infections, even at low concentrations (Kara et al., 2022).

The effects of VPS solutions on gr‐positive bacteria are summarized in Table 2. The zone diameter interpretation procedure adopted in France was employed for semi‐quantitative in vitro susceptibility testing of certain pathogenic bacteria using the agar disk diffusion test procedure (CLSI 2006). In Table 2, the Zone Diameter Control Zone Interpretive Standards (mm) for Neomycin in the three ranges of ≤12, 13–16, and ≥17, and for tetracycline in the three ranges of ≤14, 15–18, and ≥19 were reported as resistant, intermediate, and susceptible.

**TABLE 2:** Inhibition zone diameters of red fruit VPSs for different indicator microorganisms.

Indicator bacteria	Inhibition zone diameter (mm)						Antibiotic disc	
	Hawthorn	Gilaburu	Blackberry	Pomegranate	Rosehip	VPS mix	TE‐30	NE‐30
Gram‐negative bacteria								
*E.coli* RSSK 09036	14.61	18.88	19.85	15.56	18.02	15.78	12.63	22.00
*P. aeruginosa* ATCC 27853	16.25	19.28	14.15	12.66	13.34	14.88		
*S. parathypi* A NCTC13	23.15	14.38	10.38	12.21	22.45	14.20	21.39	NE
*A. baumannii*	20.21	17.20	ND	ND	10.76	18.34		
*P. putita*	15.17	16.99	13.20	11.62	11.23	11.90		
*S. Poona* RM 2350	17.47	20.15	14.39	11.78	11.12	15.88	20.00	NE
*K. pneumoniae* (ATCC700603)	16.34	9.56	11.23	10.45	12.22	15.20	12.56	20.00
*C. jejuni* ATCC33560	15.23	19.87	10.21	10.98	ND	12.25	21.00	21.00
Mold								
*Candida albicans* ATCC1023	25.32	17.66	21.19	13.22	23.22	11.97	NE	NE
Gram‐positive bacteria								
*S. aureus* ATCC 29213	23.55	20.21	18.25	11.15	22.56	14.70	15.36	20.32
*B. subtilus* B 354	18.32	18.90	16.48	12.22	18.12	14.88	22.31	12.00
*S. agalactiae*	17.65	20.19	21.35	13.08	23.01	16.20	22.34	NE
*E. faecalis*	14.77	17.80	10.23	10.88	14.77	13.11	9.53	20.00
MRSA	14.88	19.88	17.23	11.20	13.89	13.80	20.21	NE

*Note*: VPS mix was used by mixing all VPSs at a ratio of 1:1:1:1:1. Zone diameters were measured using a digital caliper. Tetracycline (TE 30) and Neomycin (N30) disks were used for antimicrobial susceptibility testing of bacterial cultures in agar well diffusion assays, and supplemental Tables CLSI procedural standards were taken as reference in the results. Additionally, petri dishes are indicated in Figure 3.

Abbreviation: NE: Not effected.

**FIGURE 3:**
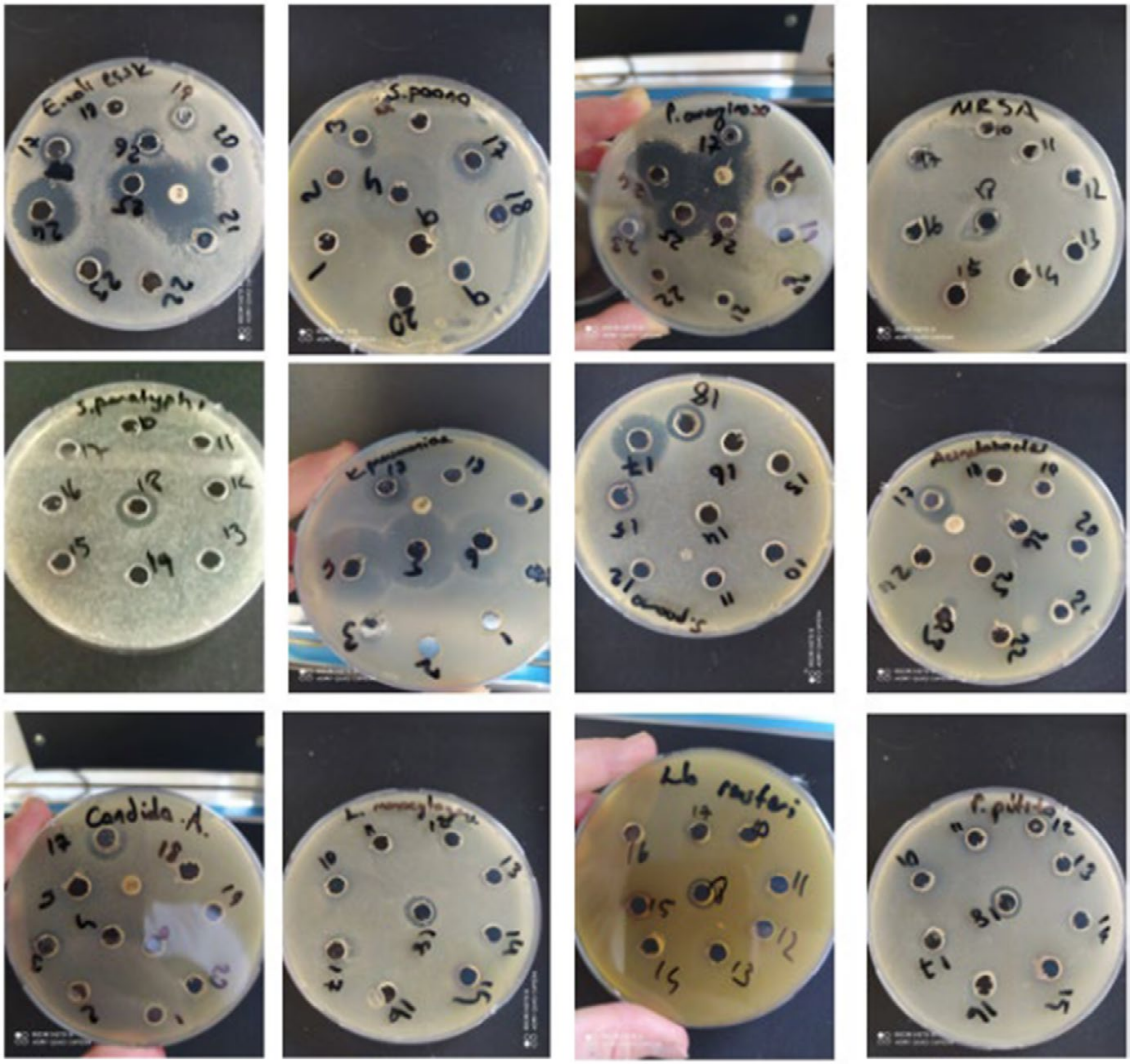
Antimicrobial activity of VPSs against gram± bacterial strains determined by well diffusion method. Representative pictures of the bacteria studied. The standardized bacterial inoculum was spread evenly on a Mueller‐Hinton Agar plate to obtain a grass culture. These were grouped according to water and ethyl alcohol extracts. 100 μL of sample (100%) was loaded.

When the VPSs were classified according to their type, the strongest antibacterial effect of hawthorn VPS was observed with *C. albicans* (zone diameter of 25.32 mm) for gr‐negative bacteria and *S. aureus* (zone diameter of 23.55 mm) for gr‐positive bacteria. The strongest antibacterial effect of gilaburu VPS was observed with *S. poona* (zone diameter of 20.15 mm) for gr‐negative bacteria and *S. aureus* (zone diameter of 20.21 mm) for gr‐positive bacteria. The strongest antibacterial effect of blackberry VPS was observed with *C. albicans* (zone diameter of 21.19 mm) for mold and *S. agalactiae* (zone diameter of 20.21 mm) for gr‐positive bacteria. The strongest antibacterial effect of pomegranate VPS was observed with *E. coli* (zone diameter of 15.56 mm) for gr‐negative bacteria and *S. agalactiae* (zone diameter of 13.08 mm) for gr‐positive bacteria. The strongest antibacterial effect of rosehip VPS was observed with *C. albicans* (zone diameter of 23.22 mm) for mold and *S. agalactiae* (zone diameter of 23.01 mm) for gr‐positive bacteria. Finally, the strongest antibacterial effect of the VPS mixture was observed with *A. baumannii* (zone diameter of 16.20 mm) for gr‐negative bacteria and *S. agalactiae* (zone diameter of 16.20 mm) for gr‐positive bacteria.

When they were compared according to their maximum antimicrobial effects on gr‐negative bacteria, it was determined that blackberry VPS (zone of diameter 19.85 mm) was the most effective against *E. coli*. Hawthorn VPS (zone diameters of 23.15, 20.21, 16.34, and 25.32 mm) was effective against *S. parathypi, A. baumannii, K. pneumoniae*, and *C. albicans* bacteria. In addition, gilaburu VPS (zone diameters of 19.85, 16.99, 20.15, and 19.87 mm) was the most effective against *P. aeruginosa, P. putita, S. Poona*, and *C. jejuni*.

According to the maximum antimicrobial effects in gr‐positive bacteria, hawthorn VPS (zone diameter of 23.55 mm) was the most effective against *S. aureus*. Rosehip VPS (zone diameter of 23.01 mm) was the most effective against *S. agalactiae*, and gilaburu VPS (zone diameters of 18.90, 17.80, and 19.88 mm) was the most effective against *B. subtilus*, *E. faecalis*, and methicillin‐resistant *S. aureus* (MRSA), respectively.

#### 3.2.1 Mortality of VPS according to MBC and MIC results

The growth concentrations of VPSs are given in Table 3. The highest inhibitory effects of hawthorn VPS on gr‐positive bacteria were observed against *S. aureus* (94.6% at 0.03 mg mL^−1^), *B. subtilus* (91.9% at 0.06 mg mL^−1^), *S. agalactiace* (90.2% at 0.06 mg mL^−1^), MRSA (84.4% at 0.03 mg mL^−1^), and *E. faecalis* (71.4% at 0.22 mg mL^−1^). The highest inhibitory effects of hawthorn VPS on gr‐negative bacteria were observed against *S. paratyphi* A (94.1% at 0.13 mg mL^−1^), *A. baumannii* (90.1% at 0.06 mg mL^−1^), *P. aeruginosa’* da (89.7% at 0.13 mg mL^−1^), *P. putita* (88.4% at 0.13 mg mL^−1^), *S. poona* (88.1% at 0.03 mg mL^−1^), *K. pneumonia* (0.06 mg mL^−1^) 87.9%, *E.coli* (87.7% at 0.13 mg mL^−1^), and *C. jejuni* (75.7% at 0.06 mg mL^−1^). Hawthorn VPS also showed an inhibitory effect on mold in *C. albicans* (90.6% at 0.06 mg mL^−1^).

**TABLE 3:** Mortality, minimum bactericidal concentration (MBC), and minimum inhibition concentration (MIC) values of red VPSs.

Types of VPS												
Indicator bacteria	Hawthorn		Pomegranate		Gilaburu		Blackberry		Rosehip		VPS mix	
	(MBC_90_) 90%	(MIC_50_) 50%	(MBC_90_) 90%	(MIC_50_) 50%	(MBC_90_) 90%	(MIC_50_) 50%	(MBC_90_) 90%	(MIC_50_) 50%	(MBC_90_) 90%	(MIC_50_) 50%	(MBC_90_) 90%	(MIC_50_) 50%
*S. aureus*	0.031	<0.03	<0.5	—	0.06	<0.03	0.25	—	0.13	0.13	<0.5	0.5
*B. subtilus*	<0.5	“	<0.5	—	“	“	0.06	—	—	0.06	“	<0.5
*S. agalactiae*	“	“	0.25	—	“	“	0.13	—	0.06	—	“	0.5
*E. faecalis*	“	“	0.13	—	0.13	“	—	0.25	0.25	—	“	“
MRSA	“	“	0.5	—	0.06	“	0.03	—	—	0.03	“	“
*E.coli*	“	“	<0.5	—	“	“	0.5	0.06	0.06	—	“	<0.06
*P. aeruginosa*	“	“	0.5	0.13	“	“	0.25	—	—	0.13	“	<0.5
*S. Paratyphi A*	“	“	0.5	—	“	“	0.5	—	—	0.03	“	0.5
*A. baumannii*	“	“	0.25	—	“	“	0.06	—	—	0.50	“	“
*P. putita*	“	“	0.25	—	“	“	<0.5	0.03	—	0.13	“	“
*S. poona*	“	“	0.13	0.03	0.03	“	—	<0.03	—	0.25	“	<0.06
*K. pneumoniae*	“	“	0.25	—	—	“	—	<0.03	—	0.13	—	0.06
*C. jejuni*	“	“	<0.5	0.5	0.03	“	—	0.5	—	0.13	—	0.5
*C. albicans*	“	“	<0.5	—	0.06	“	0.5	—	0.06	—	—	0.25

*Note*: MIC and MBC levels of postbiotics from vinegars.

Abbreviations: MBC90%, minimum bactericidal concentration; MIC50%, minimum inhibitory concentration.

For gilaburu VPS, the growth of bacteria compared with the control group is shown in Table 3. The highest inhibitory effects of gilaburu VPS on gr‐positive bacteria were observed against *S. aureus* (93.4% at 0.06 mg mL^−1^), MRSA (93.3% at 0.06 mg mL^−1^), *B. subtilus* (91.9% at 0.06 mg mL^−1^), *S. agalactiace* (92.2% at 0.03 mg mL^−1^), and *E. faecalis* (92.2% at 0.13 mg mL^−1^). The highest inhibitory effects of gilaburu VPS on gr‐negative bacteria were observed against *P. aeruginosa* (93.2% at 0.13 mg mL^−1^), *S. paratyphi* A (92.7% at 0.06 mg mL^−1^), *A. baumannii* (92.6% at 0.06 mg mL^−1^), *E. coli* (92.2% at 0.13 mg mL^−1^), *C. jejuni* (91.9% at 0.06 mg mL^−1^), *S. poona* (90.7% at 0.03 mg mL^−1^), *P. putita* (84.4% at 0.06 mg mL^−1^), and *K. pneumonia* (87.9% at 0.50 mg mL^−1^). Gilaburu VPS also showed an inhibitory effect on mold in *C. albicans* (89.3% at 0.06 mg mL^−1^).

For rosehip VPS, the highest inhibitory effects on gr‐positive bacteria were observed against *S. agalactiace* (93.7% at 0.06 mg mL^−1^) and *E. faecalis* (90.2% at 0.25 mg mL^−1^). The highest inhibitory effects of rosehip VPS on gr‐negative bacteria were observed against *E. coli* (91.7% at 0.06 mg mL^−1^), *P. aeruginosa* (87.9% at 0.13 mg mL^−1^), *P. putita* (86.8% at 0.13 mg mL^−1^), *S. paratyphi* A (86.2% at 0.03 mg mL^−1^), *K. pneumoniae* (84.9% at 0.13 mg mL^−1^), *S. poona* (76.6% at 0.25 mg mL^−1^), *C. jejuni* (59.2% at 0.03 mg mL^−1^), and *A. baumannii* (53.7% at 0.50 mg mL^−1^). Rosehip VPS also showed an inhibitory effect on mold in *C. albicans* (93.8% at 0.06 mg mL^−1^), as well as effects on probiotics in *L. plantarum* (81.1% at 0.06 mg mL^−1^) and *L. reuteri* (77.8% at 0.5 mg mL^−1^).

For pomegranate VPS, the highest inhibitory effects on gr‐positive bacteria were observed against MRSA (96.0% at 0.5 mg mL^−1^), *S. agalactia* (90.3% at 0.25 mg mL^−1^), *E. faecalis* (89.5% at 0.25 mg mL^−1^), *B. subtilus* (86.4% at 0.25 mg mL^−1^), and *S. aureus* (83.8% at 0.25 mg mL^−1^). The highest inhibitory effects of pomegranate VPS on gr‐negative bacteria were observed against *P. putita* (98.6% at 0.5 mg mL^−1^), *A. baumannii* (88.8% at 0.25 mg mL^−1^), *P. aeruginosa* (87.5% at 0.5 mg mL^−1^), *S. paratyphi* A (87.2% at 0.5 mg mL^−1^), *E. coli* (83.0% at 0.13 mg mL^−1^), *K. pneumonia* (85.9% at 0.5 mg mL^−1^), *S. poona* (81.6% at 0.13 mg mL^−1^), and *C. jejuni* (67.3% at 0.03 mg mL^−1^). Pomegranate VPS also showed an inhibitory effect on mold in *C. albicans* (85.5% at 0.5 mg mL^−1^).

For blackberry VPS, the highest inhibitory effects on gr‐positive bacteria were observed against *S. aureus* (91.3% at 0.25 mg mL^−1^), MRSA (91.0% at 0.03 mg mL^−1^), *S. agalactiace* (90.5% at 0.13 mg mL^−1^), *B. subtilus* (90.3% at 0.06 mg mL^−1^), and *E. faecalis* (38.2% at 0.13 mg mL^−1^). The highest inhibitory effects of blackberry VPS on gr‐negative bacteria were observed against *P. aeruginosa* (92.1% at 0.13 mg mL^−1^), *A. baumannii* (91.2% at 0.06 mg mL^−1^), *E. coli* (89.6% at 0.5 mg mL^−1^), *S. poona* (89.4% at 0.03 mg mL^−1^), *P. putita* (84.4% at 0.5 mg mL^−1^), *K. pneumoniae* (84.2% at 0.13 mg mL^−1^), *S. paratyphi* A (43.8% at 0.5 mg mL^−1^), and *C. jejuni* (26.7% at 0.13 mg mL^−1^). Blackberry VPS also showed an inhibitory effect on mold in *C. albicans* (93.3% at 0.5 mg mL^−1^).

For the VPS mixture, the highest inhibitory effects on gr‐positive bacteria were observed against *S. aureus* (85.6% at 0.06 mg mL^−1^), *S. agalactiace* (84.9% at 0.06 mg mL^−1^), *B. subtilus* (81.2% at 0.13 mg mL^−1^), *E. faecalis* (80.0% at 0.06 mg mL^−1^), and MRSA (75.4% at 0.06 mg mL^−1^). The highest inhibitory effects of mixed VPS on gr‐negative bacteria were observed against *P. aeruginosa* (87.9% at 0.06 mg mL^−1^), *A. baumannii* (86.8% at 0.13 mg mL^−1^), *P. putita* (83.5% at 0.13 mg mL^−1^), *S. paratyphi* A (83.4% at 0.06 mg mL^−1^), *E. coli* (80.2% at 0.13 mg mL^−1^), *C. jejuni* (75.8% at 0.13 mg mL^−1^), *S. poona* (74.9% at 0.25 mg mL^−1^), and *K. pneumoniae* (62.1% at 0.06 mg mL^−1^). The VPS mixture also showed an inhibitory effect on mold in *C. albicans* (68.9% at 0.13 mg mL^−1^).

The minimum bactericidal concentration (MBC) and minimum inhibition concentration (MIC) concentrations of five red VPSs were evaluated using the data in Table 3. The MBC values of hawthorn VPS were higher than 0.5 mL mL^−1^ for all microorganisms (except for *S. aureus*, 0.03 mL mL^−1^). The MIC values of hawthorn VPS were lower than 0.03 mL mL^−1^ in all microorganisms.

The MBC values of gilaburu VPS were determined to be 0.06 mL mL^−1^ (0.13 mL mL^−1^ for only *Ent. faecalis*) for gr‐positive bacteria and 0.06 mL mL^−1^ (0.03 mL mL^−1^ for only *S. poona*) for gr‐negative bacteria. However, the value could not be determined for *K. pneumoniae*. An MBC value of 0.06 mL mL^−1^ was calculated for mold in *C. albicans*. The MIC values of gilaburu VPS for all microorganisms were 0.03 mL mL^−1^.

Blackberry VPS had the highest MBC value for gr‐positive bacteria, 0.25 mL mL^−1^ for *S. aureus*, and the lowest MBC value for MRSA, 0.03 mL mL^−1^. MBC values of 0.5, 0.25, 0.5 0.06, and 0.5 mL mL^−1^ were found for *E. coli*, *P. aeruginosa*, *S. paratyphi* A, *A. baumannii*, *and P. putita*, respectively, but they were not found for *S. poona* or *K. pneumoniae*. The MBC value for mold in *C. albicans* was determined to be 0.5 mL mL^−1^. The MIC values were the highest for *C. jejuni*, 0.5 mL mL^−1^, and lowest for the gr‐negative bacteria.

The MBC values of rosehip VPS were determined for all gr‐positive bacteria except *E. coli*. The highest MBC values were determined to be 0.25 mL mL^−1^ for *E. faecalis* and 0.13 mL mL^−1^ for *S. aureus*, and the lowest value of 0.06 mL mL^−1^ was determined for *S. agalactiae*. Only the MBC value for *E. coli* was determined from the gr‐negative bacteria as 0.06 mL mL^−1^. An MBC value could not be determined for *P. aeruginosa*, *S. paratyphi A, A. baumannii*, *and P. putita*, but the values for *S. poona* and *K. pneumoniae* were between 0.031–0.5 mL mL^−1^. The MBC value for mold in *C. albicans* was determined to be 0.06 mL mL^−1^. The highest MIC values were 0.5 mL mL^−1^ for *A. baumannii* from the gr‐negative bacteria, followed by 0.13 mL mL^−1^ for *S. aureus* from the gr‐positive bacteria. However, the values could not be determined for *S. agalactiae*, *E. faecalis*, *E. coli*, or *C. albicans*.

The MBC values of pomegranate VPS were 0.13 mL mL^−1^ for *E. faecalis* and *S. poona*, over 0.5 mL mL^−1^ for *S. aureus*, *B. subtilus* and mold, and 0.5 mL mL^−1^ for *P. aeruginosa* and *S. paratyphi A*. The MIC values were 0.031, 0.13, and 0.5 mL mL^−1^ for *S. poona*, *S. paratyphi A*, and *C. jejuni*, respectively.

The MBC values of the VPS mixture were higher than 0.5 mL mL^−1^ for positive and negative bacteria, but no value for mold could be determined. The MIC values were the lowest (0.063 mL mL^−1^) for *S. poona* and *K. pneumoniae*, and values of 0.5 mL mL^−1^ were determined for the other microorganisms (except for *C. Albicans*, 0.25 mL mL^−1^).

An important question arising from our results is how VPS applications realize their antioxidative and antimicrobial properties in vitro applications. It is suggested that our study results are due to the anti‐inflammatory, antioxidant and antimicrobial properties of VPS active components (such as organic acids, flavanoids, phenolics). Moreover, the MIC and MBC results of VPS samples at dilute concentrations appeared to be more effective than compared to undiluted VPS samples. This can be explained by the concentration change of the solution and the physicochemical change in surface tension. Unexpectedly, according to the results obtained from the MIC and MBC tests, using VPS as a mixture did not show the expected effect compared with using it alone. As a result, it is estimated that VPSs have an antagonistic effect among themselves and their effects are weakened. The antimicrobial effect was detected most in rosehip and gilaburu VPS varieties, respectively. However, it is correct to note that the antimicrobial effects of other VPS types vary depending on the bacterial species. In addition to their therapeutic effects, the most important advantages of VPS varieties are that they can be used as low‐cost nutrients and disinfectants that can be easily obtained traditionally. In addition, although the results obtained according to the analysis are at least as effective as classical drugs, it can be said that since they are fermented products of plant origin, their side effects are almost non‐existent compared to classical drugs. As a limitation of our study, human studies could compare the therapeutic properties of postbiotics with classical drugs. However, it should be emphasized that the animal model has some disadvantages since it shows a faster effect than humans. With future in vivo and clinical studies, we can focus more on other beneficial health function of VPS. Controlled clinical studies conducted over a long period of time are needed to clarify the therapeutic properties of VPS use in patients.

## 4 CONCLUSION

When vinegar fermentation is completed, the supernatant is obtained by filtration, which is called CFS or VPS. The health benefits of vinegar have been extensively studied and used by humans since ancient times. Because of in vitro analyses, existing vinegars can be used to increase the shelf life of foods, disinfect surfaces in the health sector, and help eliminate infectious diseases and increase disease resistance. In this study, the mix of VPSs was not as effective as the individual solutions. This may be because some compounds in the mixture transformed into other substances. In addition, we observed that the five VPSs had different beneficial effects, depending on the conditions and microorganisms used. A limitation of this study is that it is not known what effects these VPSs have in vivo. For this reason, clinical studies should be conducted in animals and follow‐ups.

## AUTHOR CONTRIBUTIONS

**Oğuzhan Özdemir:** Conceptualization (equal); data curation (equal); formal analysis (equal); funding acquisition (equal); investigation (equal); methodology (equal); project administration (equal); resources (equal); software (equal); supervision (equal); validation (equal); visualization (equal); writing – original draft (equal); writing – review and editing (equal).

## CONFLICT OF INTEREST STATEMENT

The author declare no conflict of interest.

## Supporting information


Data S1.

## Data Availability

All data generated or analyzed during this study are included in this published article.
