# Supplementary material for: Vinegar postbiotic solutions obtained from five red fruits processed using traditional methods exhibit different biochemical properties and antimicrobial and antioxidant effects
Source: Food Sci Nutr. 2024 Oct 30;12(12):10136–47. doi: 10.1002/fsn3.4459 (PMC11666968; doi:10.1002/fsn3.4459)
Supplement: Supplementary file 1 — Data S1. [file FSN3-12-10136-s001.docx]

**Supplementary material 1.** Latin and commonly used names, regions in Turkey, and harvest times of fruits from which VPSs were collected

| **Fruit source of vinegars** | | | | |
| --- | --- | --- | --- | --- |
| **Latin name** | **Name** | **Harvest time** | **Origin** | **Region** |
| *Punica granatum* | Pomegranate | November | Antalya | Manavgat-Side |
| *Crataegus monogyna* | Hawthorn | November | Manisa | Turgutlu |
| *Vibirnum opulus* | Gilaburu | October | Kayseri | Bunyan |
| *Rubus caesius* | Blackberry | July | Bursa | Osmangazi |
| *Rosa canina* | Rosehip | October | Gümüşhane | Kelkit |


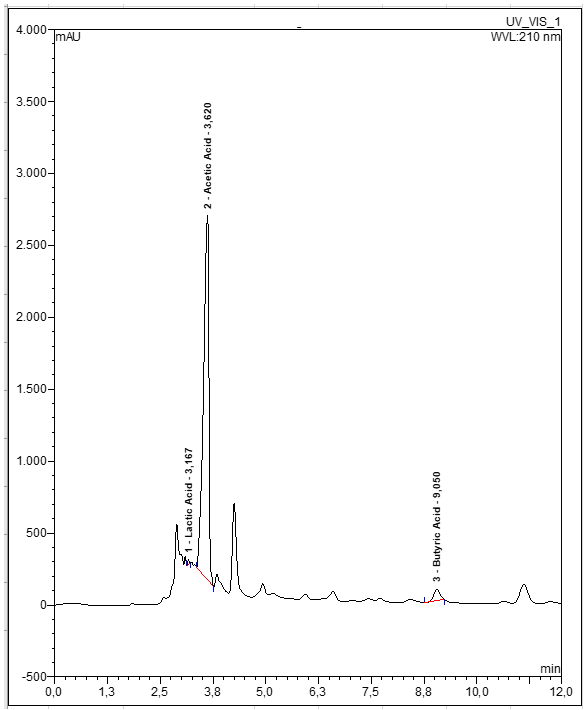


A

**Blackberry**


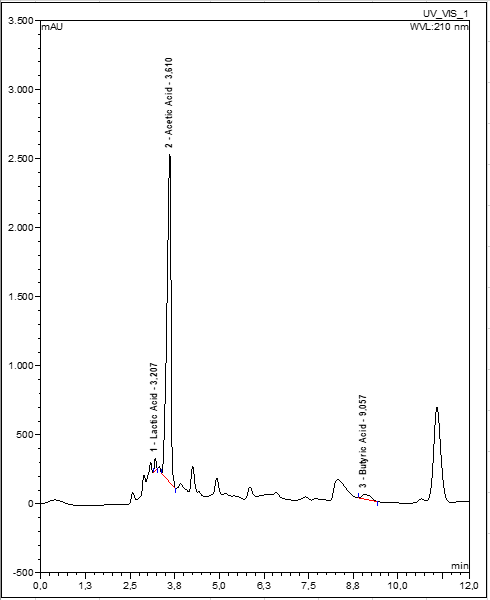


B

**Gilaburu**


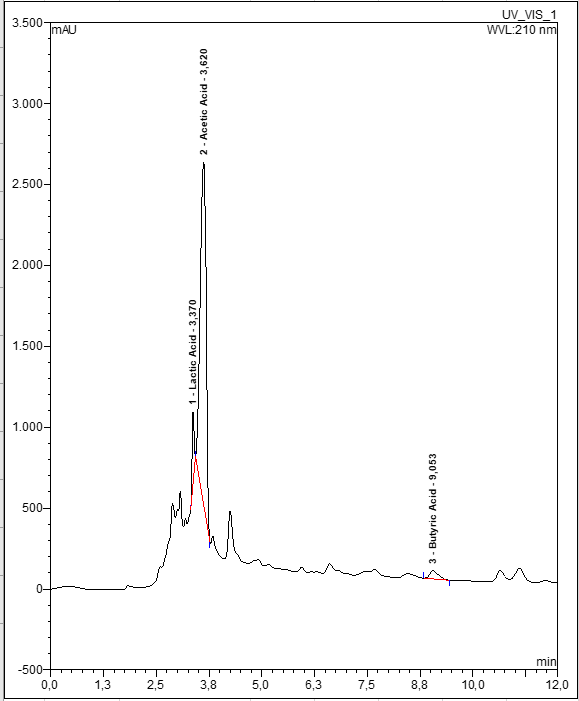


C

**Rosehip**


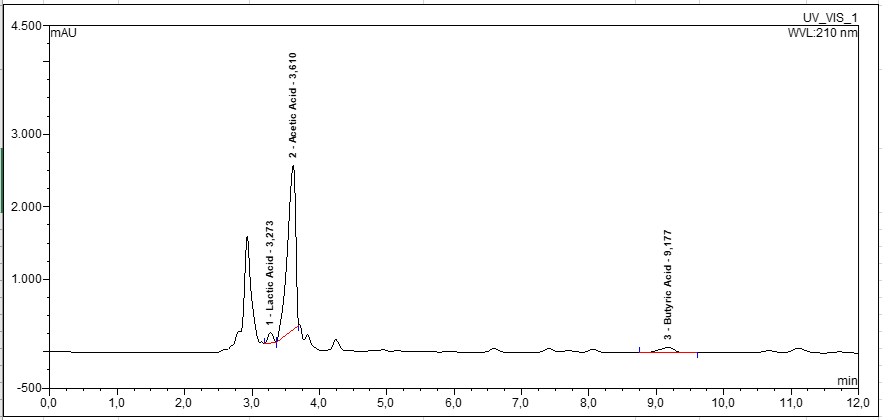


D

**Hawthorn**


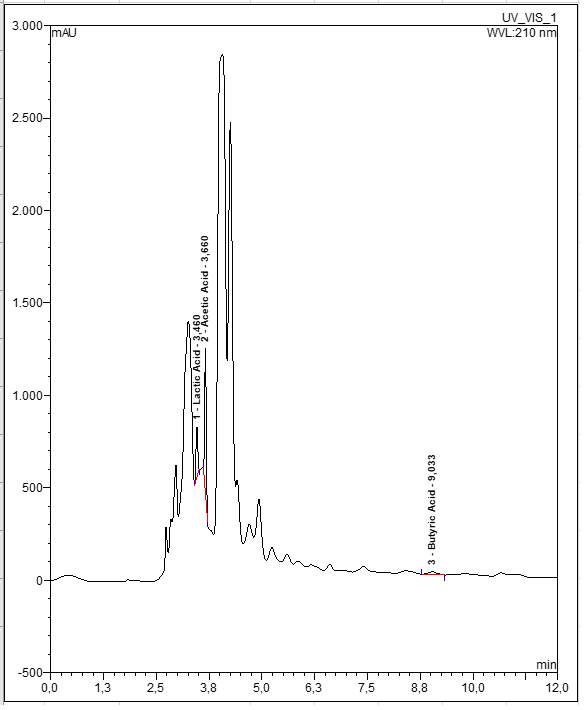


E

**Pomegranate**

**Supplementary material 2.** HPLC chromatogram images of vinegar organic acids. . HPLC-DAD (Thermo/DIONEX Ultimate 3000 series, Thermo Fisher Scientific, USA) device was used for the quantitative analysis of organic acids (Diagram A-E).
